# Multi-Organs-on-Chips for Testing Small-Molecule Drugs: Challenges and Perspectives

**DOI:** 10.3390/pharmaceutics13101657

**Published:** 2021-10-11

**Authors:** Berivan Cecen, Christina Karavasili, Mubashir Nazir, Anant Bhusal, Elvan Dogan, Fatemeh Shahriyari, Sedef Tamburaci, Melda Buyukoz, Leyla Didem Kozaci, Amir K. Miri

**Affiliations:** 1 Department of Mechanical Engineering, Rowan University, Glassboro, NJ 08028, USA; bhusal45@students.rowan.edu (A.B.); ed272@njit.edu (E.D.); am3296@njit.edu (A.K.M.); 2 Molecular Biology and Genetics, Faculty of Engineering and Natural Sciences, Istinye University, Istanbul 34010, Turkey; 3 Department of Pharmaceutical Technology, School of Pharmacy, Aristotle University of Thessaloniki, GR-54124 Thessaloniki, Greece; karavasc@pharm.auth.gr; 4 Department of Microbiology, Sher-i-Kashmir Institute of Medical Sciences, Srinagar 190011, India; mubashir.nazir28@gmail.com; 5 Department of Biomedical Engineering, New Jersey Institute of Technology, Newark, NJ 07102, USA; 6 Institute of Health Science, Department of Translational Medicine, Ankara Yildirim Beyazit University, Ankara 06800, Turkey; shf.ir65@gmail.com; 7 Izmir Institute of Technology, Graduate Program of Biotechnology and Bioengineering, Gulbahce Campus, Izmir 35430, Turkey; sedeftamburaci@gmail.com; 8 Izmir Institute of Technology, Department of Chemical Engineering, Gulbahce Campus, Izmir 35430, Turkey; 9 Care of Elderly Program, Vocational School of Health Services, Izmir Democracy University, Izmir 35140, Turkey; melda.buyukoz@idu.edu.tr; 10 Department of Medical Biochemistry, Faculty of Medicine, Ankara Yildirim Beyazit University, Ankara 06800, Turkey; dkozaci@ybu.edu.tr; 11 Department of Mechanical and Industrial Engineering, New Jersey Institute of Technology, Newark, NJ 07102, USA

**Keywords:** small drugs, microfluidics, high-throughput screening, multi-organ-on-a-chip

## Abstract

Organ-on-a-chip technology has been used in testing small-molecule drugs for screening potential therapeutics and regulatory protocols. The technology is expected to boost the development of novel therapies and accelerate the discovery of drug combinations in the coming years. This has led to the development of multi-organ-on-a-chip (MOC) for recapitulating various organs involved in the drug–body interactions. In this review, we discuss the current MOCs used in screening small-molecule drugs and then focus on the dynamic process of drug absorption, distribution, metabolism, and excretion. We also address appropriate materials used for MOCs at low cost and scale-up capacity suitable for high-performance analysis of drugs and commercial high-throughput screening platforms.

## 1. Introduction 

The failure of drug design in clinical trials is rooted in the differences between the human body and the preclinical animal models, resulting in incorrect predictions of pharmacokinetics and pharmacodynamics, such as clearance, safety margins, toxicity, and efficacy [1,2]. Clinical trials on more than 2000 drugs indicated the incompatibility of animal test results for toxic responses in the human body [3]. The growing need to develop viable in vitro alternatives to animal testing has generated the organ-on-chips technology, which combines biotechnology, cell biology, biomaterials, and biomedical sciences to recapitulate an organ or tissue microenvironment [4,5,6]. In contrast to single organ chips, which aim to recapitulate the biological function of individual organs, multi-organs-on-chips (MOCs) are introduced to integrate multiple organs on a single platform. Interconnected microchambers in organs-on-chips create dynamic drug absorption, distribution, metabolism, and excretion [4,5]. The complexity and need of multiple components, such as the liver, have led to the development of MOCs used for drug screening and toxicology testing [2,7,8,9]. The efforts associated with MOCs and how they facilitate their seamless transition from prototyping to clinical practices should be recognized to transform healthcare provision and patients’ lives.

**Figure 1:**
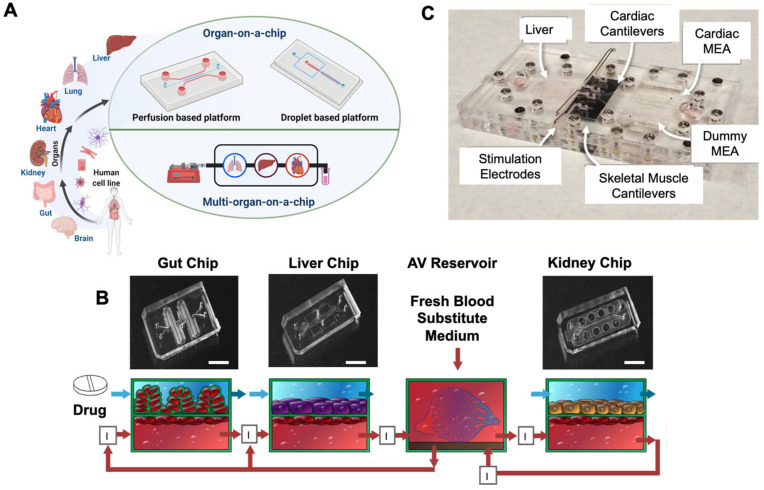
(**A**) Photographs of the microfluidic platforms. Created with biorender. (**B**) Diagrams of the gut, liver, and kidney chips fluidically linked to each other and the reservoir; bar scale is 5 mm. Reprinted with permission from [1], Spring Nature 2020. (**C**) Photograph of the multi-organ housing as an immune system featuring recirculating THP-1 immune cells with cardiomyocytes microelectrode arrays (MEA), skeletal muscle, and liver in separate compartments. Reprinted with permission from [12], John Wiley and Sons, 2020.

The main motivation behind MOCs was to include a gut part for modeling drug absorption and liver part for modeling drug metabolism. A combination of these components has been tested with a kidney model for studying drug response and its metabolism [10,11]. Pires de Mello et al. designed a heart-liver-skin MOC to investigate acute and chronic drug exposure effects on tissue function. Maschmeyer et al. developed a four-part MOC integrated with sequentially connected intestine, liver, skin, and kidney parts, which provide stable homeostasis across different organ parts to investigate the systemic toxicity for drug candidates [10]. In another study, gut-liver-kidney and bone marrow-liver-kidney MOCs were developed to predict the pharmacokinetic parameters of the orally administered drug nicotine and then intravenously injected the anticancer drug cisplatin [1]. In addition, a MOC with circulating monocytic cells as a functional human immune model was developed to analyze the tissue-specific immune responses of the cardiac, skeletal, and hepatic compartments to the anti-arrhythmic drug amiodarone [12]. Gut-on-chip was used to study the impact of the coxsackievirus B serotype 1 in the intestinal cells. The study showed the destructive effect of the virus on villi and the integrity of the whole epithelium and passage through the gut lumen into the vascular channel. The detectable cytopathic results and the increase in IL-8 implied active replication and the release of infectious virions [13,14]. The study can evaluate other enteroviruses and drug development with good precision. The chances of success are higher because of the well-understood physiology in miniaturized 3-D in vitro models (see Figure 1).

Microfluidic-based devices have been developed based on drug carrier-free and drug carrier-integrated chips, which also involve a carrier loaded with the therapeutic agent, enabling spatiotemporal control over the mobility of small-molecule drugs. Compared to conventional systems, microfluidic devices provide targeted and sustained release, thus avoiding the burst distribution of drugs that improves safety and compliance through pain reduction [15]. Modern micro and nanotechnological approaches enable spatial and temporal control over the release of drugs. We can generate tunable drugs with sustained drug release properties [16] from fibrous materials [17], microgels [18], hydrogels [19], polymeric implants [16], and DNA logic gate circuits [20]. The concept of applying microdevices for regulating the release of small molecules emerged in 1998 when Santini et al. proposed a microchip demonstrating controlled and pulsatile release of single or multiple molecules [21]. The advancements in scientific knowledge and the cross-disciplinary efforts for recapitulating experiments in more controlled conditions led to MOC developments [22,23].

The recent advances in organ-on-chips and MOCs technologies have been extensively reviewed in the literature, providing a thorough insight into the development of microphysiological systems resembling the liver and heart [24], intestine [25], lung [26], kidney [27], and multi-organ [2,28,29,30,31] functions for drug discovery and drug toxicity screening applications. Driven by the momentum of MOCs technologies, the current review will cover the latest advancements and challenges encountered in the field of MOC systems and the essential parameters for their successful and timely commercialization.

## 2. Small Molecules: Properties and Applications

Small-molecule drugs have low molecular weights (100–1000 g/mol or 0.1–1 kDa) and include chemotherapeutics, steroids, and antibiotics. These small-sized molecules can penetrate the cell membrane and modulate intracellular signaling pathways [32,33,34,35]. They can interfere with tumor-induced cell proliferation and development via interrupting various protein pathways [36]. Off-target effects and limited efficacy of drugs make small molecules preferred for their site selectivity and sustained release [16]. Different anticancer drugs have benefited from being small molecules and are applied as protein inhibitors.

MOCs have been used to study the systemic absorption and metabolism of drugs. They are integrated with micro-pumps and channels to create multi-organ models, such as the intestine, liver, skin, and kidney [37]. Wagner et al. designed a dynamic MOC by human liver and skin coculture to investigate the toxicity of troglitazone on day 6 post-incubation. Results indicated a dose-dependent response to troglitazone after the 6-day treatment [38]. In another study, Edington et al. designed an interconnected microfluidic device made of multi-cultures for modeling the gut, endometrium, lung, liver, heart, and brain for lipophilic drugs [39].

MOC designs for the blood brain barrier have come into prominence as potential models to obtain significant predictions for the transport and efficacy of nanomedicine [40]. Miller and Shuler developed a MOC model for a 13-organ system with various cell lines by mimicking the main parenchymal organs and physiological barrier tissues in the human body to investigate the inter-organ transport of biological agents for drug response [41]. Mucus is another significant biological barrier for the uptake and absorption of particulate drug carriers. Jia et al. developed a mucus-chip model to optimize the mucosal absorption of drugs by investigating the penetration and quantifying the transport of polyethylene glycols-based nanocarriers across the mucus. This mucus-on-chip enabled effective visualization and quantitative data on the absorption of drug nanocarriers during muco-penetration [15]. Drug evaluation depends on real-time monitoring of anticancer drugs in the tumor microenvironment. Tang et al. developed a biomimetic microfluidic tumor microenvironment consisting of a coculture of tumor and endothelial cells [42]. This model included a vascular part forming a total lumen under shear flow and communicating with the 3-D solid tumor part.

Besides classical microfabrication techniques, 3-D bioprinting has a high potential in MOC technology using an integrated system in dynamic conditions. In the 3-D bioprinting technique, with bioinks and a single programmable manufacturing step, the desired porosity, interconnectivity, and pore design can be easily adjusted, which is critical for tissue remodeling of the different parts of the body [43]. This technique also has the advantage of incorporating different types of biomaterials, cells, and biomolecules in a complex structure within one controllable process step [44], which makes it easy to mimic a complex organ structure.

## 3. Organs-on-Chips

### 3.1. Fabrication Methods

Conventional microfluidics fabrication methods involve the use of lithography-based molding and casting processes. The processes, such as replica molding, injection molding, and embossing, assist in fabricating MOCs. Silicon, glass, and plastic materials are used in such fabrication methods [45]. One key feature is optical transparency, which restricts the available materials and manufacturing techniques [44]. The use of polydimethylsiloxane (PDMS) leads to optical transparency, biocompatibility, flexibility, and gas permeability [46].

Among the aforementioned microfluidic fabrications processes, replica molding has been used to create PDMS microdevices containing microfabricated structures that mimic the endothelial–epithelial interface [47]. The geometry of this engineered tissue interface was enhanced to resemble the liver’s blood flow rate and provide proper orientation to rat hepatocytes (liver epithelial cells) similar to their in vivo alignment along the endothelium-lined sinusoidal barrier. Simple reconstitution of the microarchitecture of this tissue–tissue interface was sufficient to prompt cultured hepatocytes to self-organize into hepatic cord-like structures and form functional bile canaliculi in vitro even in the absence of living endothelium. Laser ablation and sacrificial replica molding techniques were applied to develop microscale 3-D collagen scaffolds replicating human intestinal villi’s geometry [48]. In this work, the culture of human Caco-2 intestinal epithelial cells produced 3-D epithelial structures exhibited by villi in the human jejunum. A microfabricated breast model was created by applying a similar kind of PDMS-based replica molding [49]. Engineered automation can resolve the precision-related and repeatability limitations of in vitro models. Automated digital microfluidics proved electro-wetting-based control of hepatic organoids in a microdevice containing a set of electrodes to enumerate the behavior of 3-D hepatic platforms in media droplets and monitor the hepatic functions [50]. The highly controlled rate of material exchange and monitoring helped achieve a fast and rapid screening of the cell–drug interactions. Table 1 summarizes some key properties of materials used for the fabrication of microfluidic devices. Silicon and glass have been the ancestors of polymer and hydrogel materials widely adopted in microfluid applications. Among the polymeric materials commonly used, Teflon is a soft, inert, and optically transparent material and relatively permeable to gases [51]. Polycarbonate is a durable, transparent, low-cost material that absorbs UV and has showed low resistance against certain organic solvents [52,53]. Styrene ethylene butylene styrene is a thermoplastic elastomer optically transparent, flexible, and adhesive with low partitioning of drugs and small hydrophobic compounds [54]. Hydrogel-based materials, such as collagen [55,56] and silkworm [57,58,59], are biocompatible and bioactive, enhancing cell attachment and proliferation. However, their poor mechanical properties and low batch-to-batch consistency may compromise experimental reproducibility and cellular response [60]. 

### 3.2. Drug Assays

Patients treated with protein-based drugs frequently develop drug-specific neutralizing antibodies, rendering the drugs unavailable to the target sites [74,75]. Neutralizing antibodies can also adversely affect other organs, as demonstrated by recombinant human erythropoietin leading to anemia resulting from antibody-dependent immune responses, which destroy both extrinsic and intrinsic erythropoietin, thus causing abnormal RBC development and production [76,77].

A study found that the effects on the potency, efficiency, hepatotoxicity, and hematological toxicity of an anticancer medicine (Tegafur) channelized with a microfluidic device that were not observed in conventional tissue culture more closely mimicked the results obtained in vivo [78]. Another study observed the previously unknown cardiotoxicity of a chemotherapeutic drug (bleomycin) because of the crosstalk between the lung and the heart tissues [79].

In vitro models provide better physiology, immune status, anatomy, drug metabolism, and host–pathogen interactions [80]. Many drugs have been withdrawn from the market after obtaining approval for humans because of their toxicity, like hepatotoxicity, liver toxicity, and cardiac toxicity (Table S1). Pergolide drug products, indicated in the treatment of Parkinson’s disease, have been linked to serious damage in the heart valves of patients [81], while rofecoxib (Vioxx), a nonsteroidal anti-inflammatory drug, resulted in a higher risk of heart attack in patients undergoing long-term treatment [81]. Both drugs have been voluntarily withdrawn from the market owing to their association with cardiac toxicity. Nefazodone, an antidepressant, and troglitazone (Rezulin), an antidiabetic medication, have been associated with acute liver injury and death and were withdrawn from the market due to increased risk of liver toxicity related to their use. The economic burden, the waste of human-derived resources, and more importantly, the toxic nature of these drugs to humans had detrimental implications on both patients and pharmaceutical companies. Therefore, the strategy has been adapted to use testing methods of drug efficacy with minimal failures.

Another set of microfluidic platforms has been used for studying host–pathogen interactions to predict drug pharmacokinetic responses in patients [1]. Hepatitis B virus (HBV) is among the significant health problems that have affected millions around the globe. A study showed the significance of using the liver-on-a-chip to image HBV interaction with hepatocytes accurately. Primary rat hepatocytes and immortalized bovine-derived aortic endothelial cells were cocultured on the opposite sides of a microporous membrane in a dual microchannel under the continuous flow of culture media. The hepatocytes maintained their polygonal morphology, physiology, division, and related markers like albumin and tumor necrosis factor for more than 20 days. The primary rat hepatocytes were successfully infected with HBV-infected adenovirus. The same model was modified using the primary human hepatocytes, maintaining their morphology and physiology for up to 26 days. The secretion of HBV core antigen and HBV DNA was detected after infection with HBV without adenovirus [82].

The ongoing COVID-19 pandemic has affected billions of lives in one way or another, calling for an adequate drug or vaccine. A human lung airway chip was developed to confront the Food and Drug Administration (FDA)-approved medications against SARS-CoV-2. This platform was created to mimic the human infection by airborne SARS-CoV-2. SARS-CoV-2 pseudo particles (CoV-2pp) carrying the SARS-CoV-2 spike proteins (a vital entry ligand) were channelized into the air channel. They were then exposed to human lung epithelial cells expressing high levels of TMPRSS2 and ACE2. The study showed the impact of amodiaquine and toremifene as potential inhibitors of SARS-COV2 in lung epithelial cells [83,84].

## 4. High-Throughput Applications and Current Challenges

The use of physiologically relevant MOC models as a screening tool for drug processes in health and disease conditions can accelerate their clinical application and the bench-to-bedside transition. Although the fast development of high-throughput screening (HST) has shown successful R&D productivity in the pharmaceutical industry and drug screening [85,86], there have still been some challenges in the process. First, a complete system of current HST technologies, including liquid handling equipment, data acquisition, extensive robotic liquid, and plate handling equipment, is expensive. The high cost of the HST platforms restricts the screening potential of small molecular targets [87]. Second, the cost of biological reagents and drug libraries is also high, and the current approaches make it challenging to reduce reagent consumption. While the volume capacity in a 384-well plate has been reduced to 100 μL, further minimization of micro-well plates is restricted due to uncontrolled liquid evaporation. The decrease of the volumes is also limited by the difficulty of dispensing tiny volumes smaller than ~1 μL [88]. Third, the failure rate in drug development is high in the clinical phase, and clinical drug development takes approximately ~63% of the total cost [85,86]. The use of appropriate cell-based assays in an early, preclinical stage is expected to provide a more efficient way to eliminate possible false leads due to low drug efficacy or high toxicity [89]. This strategy is complicated for current HST platforms because cell-based assays are more expensive and require complex liquid handling. There is a need for technology with low sample and reagent consumption, low cost, and cell friendly environment.

### 4.1. Fabrication Challenges

The majority of microfluidic platforms are dominated by the application of PDMS [90]. However, PDMS-based platforms cannot mimic complex tissue and organ architecture. The fabrication of multiple PDMS layers requires sequential integration, which is time-consuming, labor-intensive, and expensive [91]. Apart from PDMS, MOCs may also be bioprinted. This fabrication technique affects the quality and speed of fabrication of micro-tissue models [92]. Fabrication based on the use of a laser is limited by pre-application of cells, inkjet printing has low printing speed and high shear force [93], and extrusion has low printing speed and resolution.

In contrast, stereolithography has long-term cell viability concerns due to toxicity issues stemming from the use of radiation due to the application of UV sources [93]. Apart from the manufacturing techniques of MOCs, the functionalization of these platforms to mimic organs’ essential functions is crucial [40]. Mimicking these functions allows us to achieve accurate and reliable preclinical analysis.

**Figure 2:**
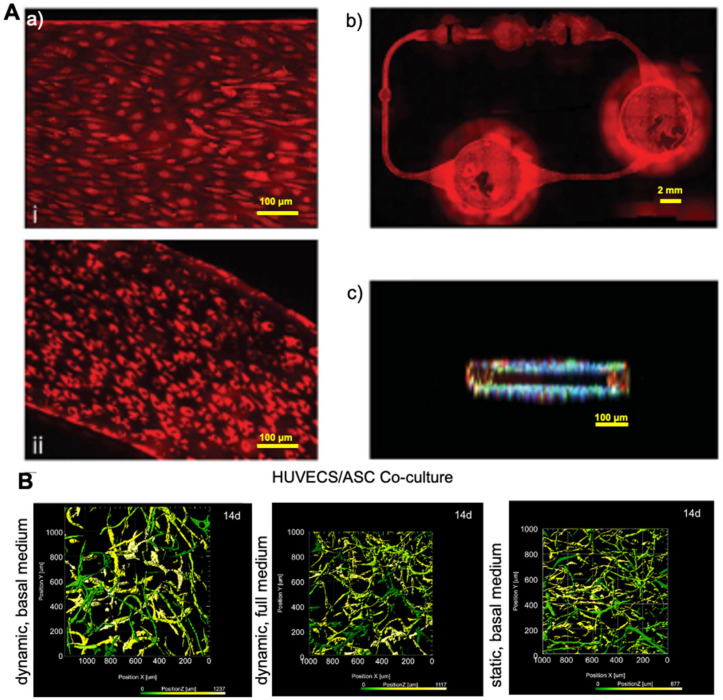
(**A**) (**a**) Characterization of the established human dermal microvascular endothelial cells (HDMECs) microvasculature at day 4, (**i**) live-cell viability staining (Calcein AM assay), (**ii**) the uniform distribution of ac-LDL uptake, (**b**) showed viable and evenly distributed HDMECs; bar scale is 2 mm, (**c**) showed HDMECs inside the microchannel. Reprinted with permission from [59], RSC 2013. (**B**) D rendering of cocultures in different configurations. Reprinted with permission from [60], Elsevier 2015.

**Figure 3:**
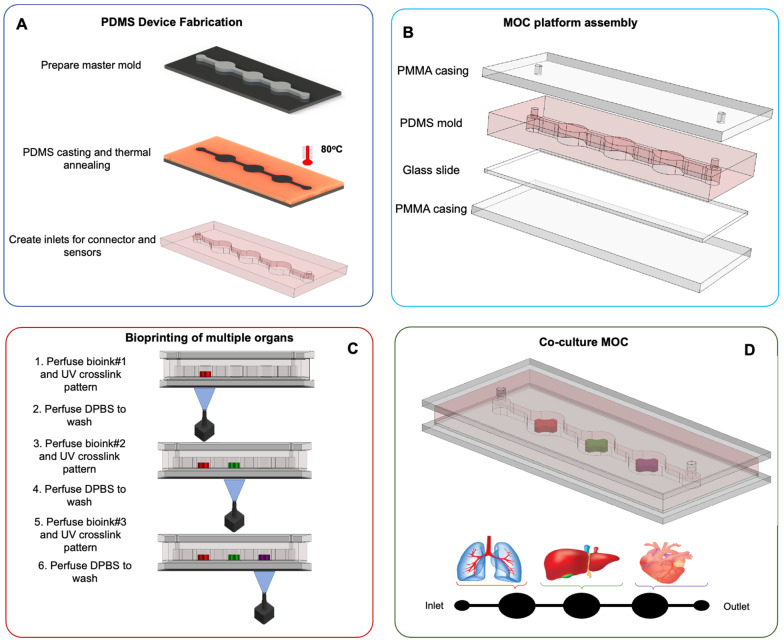
Fabrication of PDMS MOC for co-culture. (**A**) PDMS device fabrication using casting and thermal annealing. (**B**) Assembly of the MOC platform. (**C**) Bioprinting of multiple organs by applying bioink perfusion and UV crosslinking to fabricate micromodels within the culture chamber. (**D**) A potential design for the MOC platform. Created with biorender.

The vascular system allows nutrient and oxygen supply and removes metabolic waste products from the tissues while providing a selective drug barrier [94]. Recapitulating the in vivo dynamic conditions within the vascular microenvironment can be challenging, given that blood flow induces constant shear stress on vascular endothelial cells (Figure 2). In contrast, muscle cell stretching occurs during the cardiac cycle owing to blood vessel distension. The 3-D microenvironment and the dynamic mechanical events of the cardiac cycle are critical in maintaining proper vascular cell function. These factors should be taken into consideration when designing in vitro vascular platforms. The main challenges encountered during the fabrication of such platforms are related to the assembly, handling, and using conventional analytical methods [95]. It may also be challenging to adequately resemble the cylindrical geometry of vascular channels using lithographic techniques since these typically generate rectangular channels on flat surfaces [96]. Material selection may be an additional issue to consider regarding biocompatibility and compatibility with specific assays (Figure 3).

Engineering functional cardiac models that adequately recapitulate the biology of the heart is highly challenging compared to other tissues. An ideal in vitro model should mimic the heart’s cellular organization, mechanical contractions, electrical activity, and transport of molecules. Cardiomyocyte alignment within native heart tissue requires a proper design on the cell substrate [97]. The intrinsic contractions of the heart tissue are an additional challenge to be addressed since the simultaneous cardiomyocyte beating observed in vivo may be easily lost in an in vitro setup [98]. Equally crucial to the recreation of the microenvironmental cues in a cardiac construct would also be incorporating readout systems to record cardiac biological functions, such as contractility [99].

### 4.2. High-Throughput Challenges

To date, different components for cell-based microfluidic high-throughput screening (µHTS) platforms have been developed, including cell culture [88,100], introduction and transport of samples [101,102], and characterization of cell viability [103,104]. The microfluidic community has focused on the demonstration of integrating these different components into a single microfluidic device. Among current microfluidic platforms for cell-based HST, three major complementary modes of flow manipulation are perfusion flow, droplet-based, and microarray. Additionally, minimization of the well plate platform reduces reagent consumption, which is difficult due to reagent evaporation and difficulty in handling [88]. It includes expensive robotic and plate handling equipment and data acquisition systems [87], in which limitations require a microfluidic platform. The microfluidic platform provides lower reagent consumption and the ability to control cellular microenvironments [87]. The µHTS platform involves integrating numerous microfluidic components, such as valves, mixers, pumps, and sensors, to control fluid flow at the microscale [105]. The microfluidic platforms have been continuously used to detect reagents, particles, cells, or multicellular organisms for chemical and biological analysis. The detection in droplet and perfusion-based microfluidic channels is usually performed using optical, electrochemical, Raman, and mass spectrometry.

In a high-throughput screening platform, making drug gradient increases the capability of parallel screening with different drug concentrations for cell-based drug screening. The cellular response may be evaluated under multiple doses within the same platform, saving time and resources. Usually, drug gradient generators are either flow-based or diffusion-based [106]. However, flow-based gradient generators are popular among pharmaceutical evaluation. “Christmas tree structure”, a flow-based gradient generator, is well accepted for miniaturized microfluidic devices. The gradient is achieved in a miniaturized microfluidic network by sequentially diluting concentrations through a mixer [106]. The selection of the geometry for gradient generation must be considered carefully to produce an appropriate shear environment. These selections may be made by proper numerical simulation studies [107]. The simulation is helpful to determine the gradient and shear rate for individual sections with different geometry.

The optical method uses either standard microscope-based imaging or the application of fluorescence. The fluorescence allows analysis of the images, which gives an insight into biological responses. Cells can be labeled in a high-throughput screening setting, and fluorescence images may track labeled cells and micro-objects [108]. The high-throughput screening platform may integrate multiple sensors. These sensors use multiple techniques, such as conductive, resistive, capacitive, and others, to detect the presence of micro-objects and biological objects [109]. Small molecules in a microfluidic platform may be detected using Raman spectroscopy and mass spectrometry [110,111]. Raman spectroscopy uses light scattering to detect the chemical structure of the analyte, while mass spectroscopy uses the measurement of the mass-to-charge ratio of ions to detect the exact molecular weight of the analyte. The application of Raman spectroscopy is limited due to its low sensitivity; hence, an enhanced approach of surface-enhanced Raman spectroscopy may be used for the high-throughput detection of molecules [112]. Mass spectrometry is fast and has a high resolution, which is used for bioanalysis [111].

**Figure 4:**
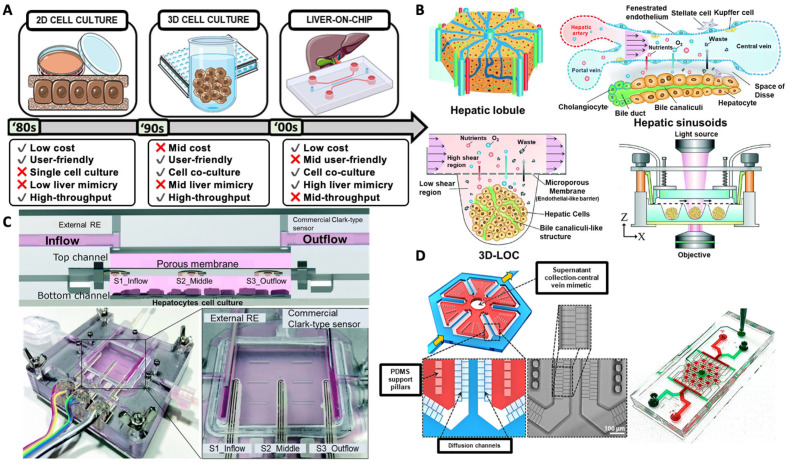
(**A**) Timeline for the cell culture technique, the advantages and limitations of 2-D culture, 3-D culture, and liver-on-a-chip. Reprinted with permission from [113], John Wiley and Sons 2021. (**B**) Perfusion culture for hepatic lobule in a concave microwell designed to mimic in the in vivo liver microenvironment and a cross-sectional view of the device. Reprinted with permission from [116], RSC 2018. (**C**) The liver platform has a cross-sectional view with an electrochemical dissolved oxygen sensor (**left**) and an accurate image of a liver system with all the fluidic and electrical connections. Reprinted with permission from [115], RSC 2018. (**D**) Schematic of a very large-sale liver-lobule-on-a-chip device fabricated using PDMS with PDMS support pillars to support the tissue chamber and align cells, and the actual device filled with food dye. Reprinted with permission from [117], IOPscience 2017.

Two-dimensional culture has been widely used for high-throughput screening, but it fails to replicate the 3-D model of the liver; thus, the 3-D culture technique was developed. A liver-on-chip platform was designed to recapitulate the native hepatic microenvironment, provide physiological fluid flow and recapitulate hepatic function (Figure 4A) [113]. The organ-on-chip has been applied to mimic rat, dog, and human liver platforms, where the cells were subjected to physiological flow. This platform allows the prediction of liver toxicity detected in animal studies (Figure 4B) [114]. The platform enables real-time monitoring using an integrated 3-D bioprinted sensor that can be used in a liver-on-chip platform. The electrochemical dissolved oxygen sensor was printed inline at multiple places to monitor oxygen concentration (Figure 4C) [115]. It captured liver function in vitro, offering a desirable biomimetic microenvironment. The study applied spheroid models, which enable a high-throughput and parallel culture in a high mass transfer and low shear for long-term perfusion culture [116]. A large-scale liver-lobule-on-chip platform was constructed in a hybrid layout with a separate seed-feed network mimicking the central vein of the liver lobule. The array allowed passage to transport by diffusion-dominated mass transport (Figure 4D) [117].

The MOC high-throughput screening is performed using a continuous-flow, droplet-based, or microfluidic array-based platform [104,118]. The droplet-based platform works according to the principles of droplet generation, where the droplets act as miniaturized reaction compartments that enclose single or multiple cells [119] that may be subjected to drug screening. The droplet-based platform quickly allows a high number of cell and droplet variants quickly but has limited cell growth in droplets. The continuous-flow platform involves controlling the laminar flow of reagent/media to control the environmental condition. The control inflow regulates dissolved gas, temperature, pH, and shear stress inside the microfluidic platform [120]. The application of a continuous-flow platform is limited by swelling when exposed to strong solvents, a limited number of the tested material, and the possibility of cross-contamination. The array-based microfluidic platform is more compatible with drug screening since it enables parallel analysis [121] in a high-throughput approach. It also provides many samples to analyze the biology and small-molecule screening [122]. However, a microarray is limited by the inefficient removal of reagents within the reaction chamber and requires minimal volume detection.

#### 4.2.1. Liver Platforms

The liver is the largest organ in the body consisting of several cell types and the most complex function [90]. Recapitulation of the liver’s structural, cellular, and localized hemodynamic complexity is one of the major challenges in developing organotypic in vitro liver models [123,124]. Even though primary hepatocytes have been the gold standard in liver chips, their dedifferentiation in vitro has led to the use of alternative cell sources, such as mutable human embryonic stem cells and iPSCs. The first steps in attaining structural biomimicry have been made with bile tube simulation, yet functional biomimicry, such as bile acid secretion, is still in progress. The lack of testing standards, uniformity, real-time monitoring, and the relatively low throughput of liver chips are additional technical challenges to be resolved with the integration of biosensors within liver devices [125].

Drug metabolism and detoxification processes are additional challenges to be addressed in the liver. As a potential drug carrier, the metabolic fate of nanoparticles and their effect on human tissues has been intensively investigated. Within this context, Esch et al. assembled a chip based on the coculture of enterocytes, Caco-2, and mucin-producing HT29-MTX cells, and HepG2/C3A liver cells to mimic the oral uptake of carboxylated polystyrene nanoparticles (50 nm) and their effects on the liver. The Caco-2/HT29-MTX coculture posed an effective barrier to nanoparticle permeability across the cell layer, yet the nanoparticle fraction crossing this layer mediated the release of aspartate aminotransferase, an indicator of liver cell injury [126].

Liver models have been made by 2-D planar culture, matrix-less and matrix-dependent 3-D culture, layer-by-layer deposition, 3-D bioprinting, microarray, and hanging drops [125]. These fabrication strategies are viable from short- to long-term biological studies. Among these strategies, 2-D culture, 3-D-based bioprinting, and microarray strategies may be used for high-throughput screening. These strategies allow the analysis of biological responses to drugs. Still, they are limited by applying multiple cells in 2-D culture, resolution, and control over individual cells in bioprinting, and lack of spatial distribution and cellular interaction in microarray strategies.

#### 4.2.2. Lung Platforms

Lung chips have introduced a credible alternative to 2-D cell cultures, emulating the microarchitecture and the primary physiological functions of the human lung better to understand the physiology and pathology of the human lungs and perform drug screening and toxicological studies [23]. One of the major challenges of microfluidic lung platforms in their current form is their short life span, owing to their short-term compatibility. The design of more biorelevant blood flow networks could potentially minimize the foreign body response, thus providing lung devices for more long-term applications [127].

Benam et al. engineered a human lung small airway chip to recapitulate the features of asthma and chronic obstructive pulmonary disease (COPD) in vitro and evaluate the therapeutic response in the small airway chips after drug treatment to overcome the challenge of extending the drug’s residence. The PDMS microfluidic device was constructed using soft lithography and consisted of two channels separated from a polyester membrane coated on both sides with type I collagen. Primary human airway epithelial cells and primary human lung microvascular endothelial cells were cultured on opposite sides of the membrane. Epithelium exposure to interleukin-13 and substituting human airway epithelial cells with epithelial cells from individuals with COPD generated in vitro models of asthma and COPD, respectively. This represents a challenge for the small airway chip device, providing a complementary approach to in vivo models by adequately recapitulating the in vivo organ-level therapeutic responses [128].

Aiming to simulate the lung tumor microenvironment for chemotherapeutic drug screening applications, a lung ToC device was constructed using an electrospun PLGA membrane as a substrate for cell culture [129,130]. Exposure of the coculture of human lung non-small cell lung cancer cell line A549 and human fetal lung fibroblast HFL1 cells to the epidermal growth factor receptor EGFR-targeted drug gefitinib showed A549 cell resistance. In contrast, the triple coculture of A549, HFL1, and HUVEC cells resulted in the A549 cells showing a strong invasive ability and possibly causing HUVECs’ death [129]. In a similar approach, a PLGA nanofiber/PDMS composite membrane-sandwiched microdevice enabled better simulation of the tumor lung microenvironment from a biophysical and biochemical perspective, allowing for fluid perfusion under very low shear stress and cell culture under both normoxic and hypoxic conditions.

### 4.3. Tumor Platforms

ToC is essential for developing functional, reproducible, and robust microfluidics suitable for drug screening assays. This was exemplified in a colon cancer micro-tumor model perfused with the anticancer compounds fluorouracil, vincristine, and sorafenib compared with conventional monolayer cultures of endothelial or tumor cells. Results indicated that perfusable vascular networks are critical for drug safety evaluation, highlighting an additional challenge since this model better mimics the physiological microenvironment than 2-D cell cultures [131].

Identification of optimal chemotherapeutic drug combinations is sometimes essential to treat invasive carcinomas, such as bladder cancer. The therapeutic potential of a single or a variety of chemotherapeutic agents has been assessed in a microfluidic platform combining the coculture of the bladder carcinoma cell line T24 with HUVECs and drug transport across the endothelial monolayer. Results showed that even though complete inhibition of T24 cell dispersal was attained in monoculture and the presence of all four tested drugs at a concentration of 10 mM, the same effect was not observed in the T24 and HUVECs coculture. The microfluidic platform could help define combination chemotherapeutic strategies that present a significant challenge in vitro to treat aggressive tumors [132]. The non-specificity of current chemotherapeutic approaches is an additional challenge to be addressed. Towards this direction, a ToC device using a coculture of human breast cancer cells and hepatic cells aimed to identify optimum drug concentrations of doxorubicin, cisplatin, and paclitaxel that could increase drug efficacy against cancer cells and reduce toxicity against healthy cells [133].

Many improvements have been made in treating various tumor types by researchers and clinicians. However, most tumor models have poor approximations to patients’ tumors, and medications with promising tumor model data may still fail in clinical trials [134]. The complex tumor microenvironment (TME) includes different cell types, ECM-derived physical stresses, oxygen, nutrient, and biochemical gradients supported by a complex vascular web. Conventional 2-D models cannot mimic the interactive relationships among these complicated relationships between TME components, such as tumor cells, stromal cells, and physical and biochemical factors inside living tumors in the presence of blood vessels [92,135]. Animal tumor models have provided valuable insights into our basic understanding of tumor biology; however, these models cannot reflect pathogenic processes in humans due to genetic alterations [136,137]. We still need better tumor models to fully understand tumor behavior and the TME, including the inflammatory factors, immune system response effectors and suppressors, cell heterogeneity, and tumor vasculature, which is significantly different from healthy vasculature. Some models have been used to assess chemical cues that influence cell migration and invasion through a porous membrane that separates two chambers in Transwell containers [138]. Multicellular tumor spheroids can mimic cell–cell and cell–ECM interactions between tumor cells and the TME. Spheroids may develop oxygen and nutrition gradients, resulting in an established necrotic core similar to poorly vascularized tumor tissue [139,140]. In vitro spheroids still lack the tumor vasculature in the TME.

**Figure 5:**
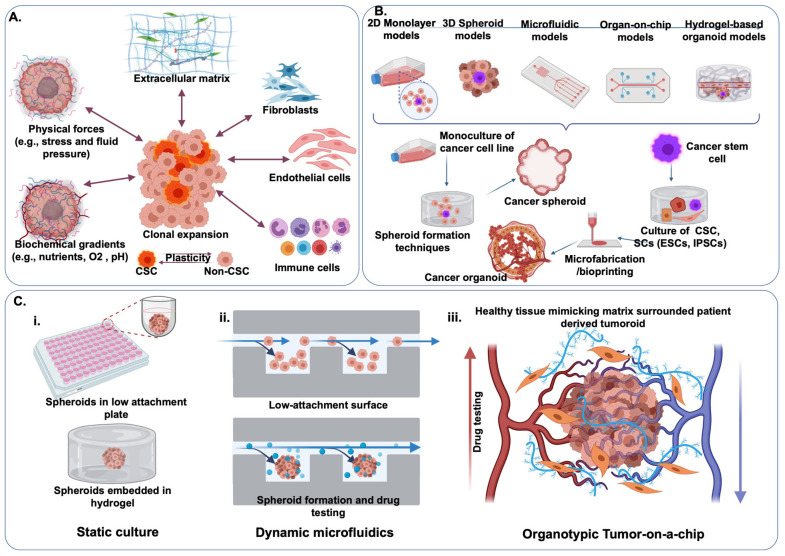
Recapitulation of the tumor microtissue with various degrees of sophistication. (**A**) Schematics TME factors and (**B**) evolution and future directions of cancer modeling from simple 2-D cultures to tumoroid models. Reprinted with permission from [150], Adv. NanoBiomed Res 2021. (**C**) The evolution of different tumor microtissue research tools, (**i**) from static tumor spheroid culture, (**ii**) dynamic microfluidics culture, and (**iii**) vascularized organotypic ToCs. Created with biorender.

Since establishing the first human cancer cell line HeLa, cancer disease models clearly illustrate an evolving nature from 2-D simple models to complex hydrogel-based organoid models (Figure 5B). Microfluidics technology has been considered a gamechanger in tumor modeling because manipulating a few chemical agents has allowed researchers to preserve tumor cells in a controlled encasement while the flow of the culture medium imposes the dynamic physiological factors in the vasculature. Researchers use soft lithography or 3-D printers to create 3-D channels and chambers in PDMS or other flexible polymers. The micro-channels are connected in micro-chambers, where cells can be captured and aggregated. Hydrogels containing cells or fluid are perfused into the built-in channels and chambers to simulate the ECM cues. By continuous perfusion of nutrients and oxygen and the removal of waste products, microfluidic devices can imitate the in vivo fluid dynamics of the TME. The interstitial pressure, soluble factor gradients, and oxygen tension are all physicochemical parameters that microfluidic devices can precisely control [141]. ToC has been developed for low-cost and high-throughput anticancer drug screening in precision medicine [142].

To investigate cancer cell migration and invasion and extracellular signaling, as well as chemotherapy and immunotherapy resistance mechanisms, multiple 3-D tumor-on-a-chip models have been demonstrated to replicate diverse forms of solid and liquid TMEs, including different stromal components, immune suppressor cells, and chemokines [143,144,145,146]. Cui et al. [147] reported a glioblastoma (GBM) on-chip platform mimicking the several GBM tumor niche subtypes (proneural, classic, and mesenchymal). The chip consists of a peripheral channel. ECs formed a vascular channel, an intermediate tumor-stromal zone, where patient-derived GBM cells, tumor-derived macrophages (TAMs), and T-cells can interact with a central port. They demonstrated an immunosuppressive tumor microenvironment analyzing cytotoxic activity and significantly reducing T-cell activation and cytotoxic function in GBM tumor niche subtypes. Similarly, Jerkins et al. [147] demonstrated a tumor spheroids model to investigate the PD-1 blockade profile by recapitulating the native tumor immune microenvironment. They showed that a short-term organotypic tumor spheroid microfluidic model could recapitulate response to PD-1 blockade and identify specific interventions that counteract resistance.

Multicellular aggregates and tumor aggregates, namely spheroids, have been used as tumor models over the years under static conditions [148]. However, these models have failed to capture the effect of blood flow. Vascularized tumor models are highly promising to mimic a hostile TME that leads to cancer progression and drug resistance. Nashimoto et al. [149] demonstrated the importance of flow in a vascular network in TME to evaluate tumor activities as a drug screening platform. They fabricated a tumor-on-chip platform that enables monitoring and assessing tumor response with intraluminal flow combining the PDMS microfluidics with multicellular tumor spheroid-embedded hydrogel. They showed that drug administration under perfused conditions did not demonstrate the dose-dependent effects of anticancer drugs on tumor activities compared to the results under static conditions.

## 5. Conclusions

There are real grounds for optimism that MOC systems will provide a more effective, time-, and cost-efficient surrogate to current small-molecule screening methods. The potential to outgrow conventional in vitro cell culture and animal tests will significantly contribute to the ‘3Rs’ regarding the use of laboratory animals. Despite the immense potential of MOCs, this technology is still at an infant stage, facing several fundamental and translational challenges that need to be addressed. The transition from MOC to human body-on-a chip platforms is the next goal to provide even more physiologically relevant systems for drug development and screening assays. The simulation of fluid flow dynamics, selecting appropriate growth factors, and culture media are important. New culture techniques are required to support the growth and proliferation of different cell types in a single platform. Increasing the relatively low throughput and automation of current MOC platforms is also essential for accelerating the dynamics of this technology. Scaling up and simplifying the complex MOC setup would make them more user-friendly and bring them one step closer to commercialization. Towards this direction, developers should also opt for platform flexibility for MOCs to be easily integrated with different platforms and efficiently operate in various applications [151]. The establishment of specific performance standards is another aspect to be considered, as MOCs are increasingly entering the market. The lack of a universal standard is one of the main reasons pharmaceutical industries are still reluctant to introduce MOCs to their portfolios. Validation of biological, technical, and analytical aspects in the performance of MOCs is critical as they can not only reproduce the organ or tissue of interest but also consistently respond to compounds with a well-established mechanism of action or toxicity. Within this context, MOCs could prove a useful preclinical tool in screening drug toxicity and efficacy and could potentially substitute animal models and support the findings of phase I and phase II clinical trials. At the same time, the use of patient-derived cell sources could additionally introduce the element of personalization. However, it is critical to establish functional and dynamic collaborations between the academic, industrial, and regulatory sectors to bridge the existing translational chasm.

The FDA, the US National Institutes of Health (NIH), and the Defense Advanced Research Projects Agency (DARPA) have acted on this demand by providing funding opportunities for the development of organs-on-chips and the regulatory support required to set the roadmap towards market [152]. In Europe, the human Organ and Disease Model Technologies (DMT) consortium recently joined forces with 21 companies, three knowledge institutions, and two foundations to develop a flexible ‘SMART Organ-on-Chip’ under the auspice of an NWO ‘Perspective’ grant. The Asia-Pacific region is witnessing exponential market growth rates, mainly due to the government funding opportunities provided to research institutes, especially in China, and the increasing number of clinical trials based on cell studies [147]. The startup founding dynamics, especially within academic settings, is also a striking example of the continuous and comprehensive efforts to bring the MOCs technology to the market. Emulate is a microfluidics startup founded in the Wyss Institute for Biologically Inspired Engineering in Harvard that has established collaborations with AstraZeneca and Johnson & Johnson to enable more precise predictions of the effects of drugs on humans. InSphero, created at the Zurich university in Switzerland, aims to develop safer and more efficient drugs. AxoSim (Tulane University), TaraBiosystems (Columbia University, New York, NY, USA), and Nortis Bio (University of Washington, Washington, DC, USA) focus on the development of nerves-on-chip, heart-on-chip, and kidneys-on-chip devices, respectively. At the same time, TissUse (Technische University, Berlin, Germany) has recently introduced a MOC with four organs integrated on the same chip, further aiming to develop a human-on-chip with 10 organs. It, therefore, is clear that only through coordinated efforts from all stakeholders will the MOC technology flourish and find its place among well-established models and procedures for small-molecule drug testing.

## Figures and Tables

**Table 1:** Biomaterials used in the fabrication of microfluidic devices.

Material	Relevant Property	Application	References
Teflon	Ease of fabrication with maximum chemical resistance	Very sensitive assays, ultra-clean tools, valves, and pumps fabrication	[51]
Acrylonitrile butadiene styrene	High resolution, best topography	Crafting of the master mold, study of pathogenic organisms	[52]
Styrene ethylene butylene styrene	Low drug absorption, optical transmittance	Human lung epithelial cells, human umbilical vein endothelial HUVECs, human alveolar epithelial cells	[54]
Chitosan	Biocompatible, effective control of stereochemistry	Biosensors, film organization	[55,56]
Silkworm	Biocompatible, pliable	Fabrication of microfluidic platforms	[57,58]
PDMS	Good turnaround time, multi-material printing, long-lasting and high-temperature-resistant substance	Master molding	[59]
Agarose	Minimal toxicity, biodegradability, tunable stability at a lower solid ratio	Chondrocytes, AML-12 murine hepatocytes, sensors, and actuators	[61,62]
Photocurable resin/polymer	Effective resolution with small characters	Study of cell growth	[63,64]
Polyurethane-methacrylate	Economical to manufacture, biocompatibility, no cytotoxicity, strong electroosmotic mobility	Increased-aspect-ratio microstructures	[65,66,67]
Polyhydroxyalkanoates	Biocompatibility, tunable, biodegradability	Microfilm barrier for vapor and oxygen	[68]
Polyethylene glycols	Cheaper than many of the material, different weight categories are available, biocompatible, cytotoxicity approximately naught	Microfluidic valves, microfluidic channels with an increased expiry time	[69]
Gelatin methacrylate	Photopolymerizable, porous membrane	Mechanistic vascular and valvular biology cell support matrix	[70]
Polylactic acid and polyglycolic acid	Mechanical biodegradation	Porous scaffold for cell culture with better adhesion	[71]
Synthetic hydrogels	Induration and contraction act as sensors and actuators	Self-regulating valves, micro-lens arrays, drug release, antigen adsorption flow sensors pH regulators	[72,73]

## Data Availability

Not applicable.

## Supplementary Materials

The following are available online at https://www.mdpi.com/article/10.3390/pharmaceutics13101657/s1, Table S1. Drugs that have been withdrawn from the market owing to their toxicity.
