# Supplementary material for: Multi-Organs-on-Chips for Testing Small-Molecule Drugs: Challenges and Perspectives
Source: Pharmaceutics. 2021 Oct 11;13(10):1657. doi: 10.3390/pharmaceutics13101657 (PMC8540732; doi:10.3390/pharmaceutics13101657)
Supplement: Supplementary file 1 [file pharmaceutics-13-01657-s001.zip › pharmaceutics-1384243-supplementary.pdf]

pharmaceutics

Supplementary Materials: Multi-Organs-On-Chips for Testing Small-Molecule Drugs: Challenges and Perspectives

Berivan Cecen, Christina Karavasili, Mubashir Nazir, Anant Bhusal, Elvan Dogan, Fatemeh Shahriyari, Sedef Tamburaci, Melda Buyukoz, Leyla Didem Kozaci and Amir K. Miri

Table S1. Drugs that have been withdrawn from the market owing to their toxicity.

Drug	Toxicity type	Indication	Withdrawn Year	Ref
Pergolide	Cardiac toxicity	Parkinson's neurodegeneration	2007	[1]
Adderall-XR	Cardiac toxicity	Attention-deficit hyperactivity disorder	2005	[2]
Rofecoxib (Vioxx)	Cardiac toxicity	Acute and chronic pain	2004	[1]
Levacetylmethadol	Cardiac toxicity	Treatment of opioid addictions	2003	[3]
Nefazodone	Liver toxicity	Depression	2003	[3]
Droperidol	Cardiac toxicity	Premedication for anesthesia	2001	[3]
Cisapride	Cardiac toxicity	Gastrointestinal reflux	2000	[1]
Troglitazone (Rezulin)	Liver toxicity	Anti-inflammatory and anti-diabetic	2000	[1]
Terfenadine	Cardiac toxicity	Allergies	1997-1999	[1]

References

1. Z.P. Qureshi, E. Seoane-Vazquez, R. Rodriguez-Monguio, K.B. Stevenson, S.L. Szeinbach, Market withdrawal of new molecular entities approved in the United States from 1980 to 2009, Pharmacoepidemiol. *Drug Saf.* **2011** 772–777. https://doi.org/https://doi.org/10.1002/pds.2155.
2. L.M. Wang, M. Wong, J.M. Lightwood, C.M. Cheng, Black Box Warning Contraindicated Comedications: Concordance Among Three Major Drug Interaction Screening Programs, *Ann. Pharmacother*. **2010**, 28–34. https://doi.org/10.1345/aph.1M475.
3. R. McNaughton, G. Huet, S. Shakir, An investigation into drug products withdrawn from the EU market between 2002 and 2011 for safety reasons and the evidence used to support the decision-making, *BMJ Open*. **2014**, e004221. https://doi.org/10.1136/bmjopen-2013-004221.
